# How the oxygen tolerance of a [NiFe]-hydrogenase depends on quaternary structure

**DOI:** 10.1007/s00775-015-1327-6

**Published:** 2016-02-09

**Authors:** Philip Wulff, Claudia Thomas, Frank Sargent, Fraser A. Armstrong

**Affiliations:** Department of Chemistry, University of Oxford, Oxford, UK; Division of Molecular Microbiology, School of Life Sciences, University of Dundee, Dundee, DD1 5EH UK

**Keywords:** Electron transfer, Iron-sulfur clusters, Quaternary structure, Hydrogen, Hydrogenase

## Abstract

‘Oxygen-tolerant’ [NiFe]-hydrogenases can catalyze H_2_ oxidation under aerobic conditions, avoiding oxygenation and destruction of the active site. In one mechanism accounting for this special property, membrane-bound [NiFe]-hydrogenases accommodate a pool of electrons that allows an O_2_ molecule attacking the active site to be converted rapidly to harmless water. An important advantage may stem from having a dimeric or higher-order quaternary structure in which the electron-transfer relay chain of one partner is electronically coupled to that in the other. Hydrogenase-1 from *E. coli* has a dimeric structure in which the distal [4Fe-4S] clusters in each monomer are located approximately 12 Å apart, a distance conducive to fast electron tunneling. Such an arrangement can ensure that electrons from H_2_ oxidation released at the active site of one partner are immediately transferred to its counterpart when an O_2_ molecule attacks. This paper addresses the role of long-range, inter-domain electron transfer in the mechanism of O_2_-tolerance by comparing the properties of monomeric and dimeric forms of Hydrogenase-1. The results reveal a further interesting advantage that quaternary structure affords to proteins.

## Introduction

**Fig. 1:**
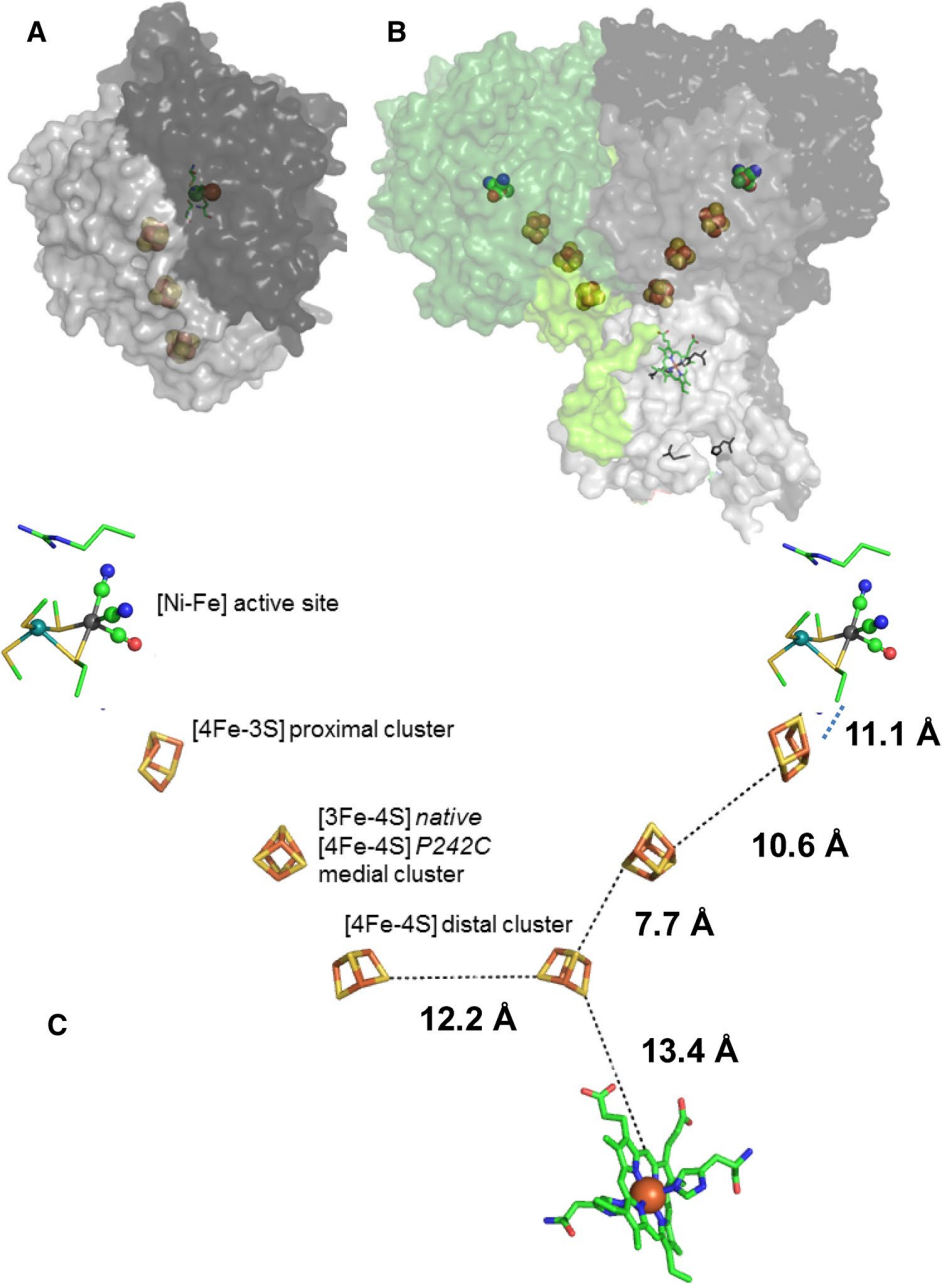
Views of [NiFe]-hydrogenases of relevance to this paper. **a** The (αβ) monomeric [NiFe]-hydrogenase from *D. fructosovorans* hydrogenase. **b** The (αβ)_2_ dimeric [NiFe]-hydrogenase from *E. coli* (Hyd-1). This structure (PDB: 4GD3) of the P242C variant with a P-to-C exchange at the medial FeS cluster also shows a cytochrome subunit that is absent from other crystal structures. **c** The redox centers in Hyd-1: [NiFe] active site and iron sulfur clusters labeled with edge-to-edge electron transfer distances. The position of the *b*-type heme in the membrane anchor is also shown (from the structure of P242C)

Hydrogenases, microbial metalloenzymes that produce or oxidize H_2_, are important for understanding the energy balance and metabolism of microoganisms, in addition to being inspirational models for future hydrogen catalysts [1, 2]. The active sites of the two main classes, known as [NiFe] and [FeFe]-hydrogenases, contain the metals Ni and Fe (as in [NiFe]-hydrogenases) or just Fe (as in [FeFe]-hydrogenases) in unusual coordination shells that have a [Fe(CO)(CN)(RS)] subcomplex as a minimal motif. Figure 1 provides structural information that will be relevant for this paper. So-called ‘standard’ [NiFe]-hydrogenases consist of a large (*α*) subunit containing the [NiFe]-active site and a small (*β*) subunit containing a relay of FeS clusters to transfer electrons. These air-sensitive enzymes have been extensively investigated by crystallography and spectrosocopic techniques with significant input from molecular biology strategies [2]. Attached to an electrode, hydrogenases are extremely efficient electrocatalysts, and protein film electrochemistry (PFE), in which catalytic rate is measured directly as current, has made important contributions to our understanding of their functional properties [3–5].

A special sub-category of [NiFe]-hydrogenases, known as O_2_-tolerant hydrogenases, have the special property of displaying sustained activity in the presence of O_2_ [6–10]. These hydrogenases operate because they destroy O_2_ by rapidly converting it to water, thus avoiding reactive oxygen species that would otherwise oxygenate the active site and render it inactive for long periods—a state/states known as ‘Unready’ or ‘Ni–A’ [10–12]. Under H_2_, membrane-bound O_2_-tolerant hydrogenases react with O_2_ to form a Ni(III)-OH complex, known as Ni–B (‘Ready’), that is rapidly re-activated by reduction to an active Ni(II) species with release of the OH ligand. An efficient electron supply thus serves two roles—(a) securing complete reduction of O_2_, by-passing reactive intermediates, and (b) ensuring rapid re-activation of Ni–B (regarded as a resting state). The structures of ‘standard’ (strongly O_2_-inhibited) [NiFe] hydrogenases, such as *D. fructosovorans* hydrogenase (Fig. 1a) [1, 13], have been known since 1995, but only recently have the structures of O_2_-tolerant hydrogenases of the subgroup of membrane-bound respiratory [NiFe]-hydrogenases (MBH) isolated from *Ralstonia eutropha* H16, *Hydrogenovibrio marinus*, *Escherichia coli* and *Salmonella enterica*, been established [14–17]. These new structures have not only revealed a novel [4Fe-3S] cluster, able to transfer two electrons sequentially [9, 15–18], but they also show interesting quaternary organization that may also be important in conferring O_2_-tolerance, as discussed below. The MBHs not only contain additional membrane-domain subunits that dissociate upon isolation with detergents, but also exist as oligomers of the minimal αβ ‘heterodimer’.

The crystal structure of Hydrogenase-1 (Hyd-1) from *E. coli* shows it to be a (αβ)_2_ dimer, in contrast to the (αβ) monomer of standard hydrogenases (Fig. 1a, b) [16]. The structure supports earlier determination of the molecular mass of Hyd-1 as 200 ± 20 kDa, [19]. It was also known that in vivo, Hyd-1 associates with membrane-intrinsic subunits that contain cytochrome *b* [19, 20]. Although the first crystal structure obtained for Hyd-1 did not include its cognate cytochrome *b* subunit, a subsequent structure obtained for P242C variant (depicted in Fig. 1b) included the membrane-intrinsic subunits in which one cytochrome *b* remained attached [21]. In the isolation of solubilized Hyd-1, the cytochrome *b* subunits are normally lost during the homogenization stage.

The distance between the two distal clusters in each half of Hyd-1 is only 12.2 Å. It has been proposed that an inter-site distance below 14 Å is generally short enough to allow electron tunneling to occur at a sufficient rate that catalysis is not limited [22]. Comparing the distances between the other iron-sulfur clusters in Hyd-1 (ca. 12.2 vs. 7.7 Å, 10.6 and 11.1 Å Fig. 1c) therefore suggests that electron exchange between the two distal clusters and thus between the two (αβ) halves should be feasible. Shomura and co-workers, having solved the similarly heterotetrameric structure of the membrane-bound hydrogenase (MBH) from *H. marinus*, ruled out the possibility that dimerization might be an artifact of crystallization, on the basis that the contact between heteromers adds up to 11 % of the total surface area of the protein and the subunits have precisely matching orientations—the latter observation pointing towards purposeful joint alignment on the cell membrane [15].

In an unusual experiment that shed further light on the importance of long-range intermolecular/interfacial electron transfer in O_2_ tolerance, Wait and co-workers measured the power characteristics of a membrane-less fuel cell with two carbon electrodes, one modified with Hyd-1 (the H_2_-oxidizing anode) and the other with bilirubin oxidase (the O_2_-reducing cathode) [23]. Such an investigation is an electrochemical experiment without a source of potential control or external electrons. The O_2_-tolerant hydrogenase allows the fuel cell to operate with a non-explosive H_2_-air mixture; however, use of a weak H_2_ mixture (3 %) resulted in loss of power, apparently irreversibly, when the load resistance was set sufficiently small to collapse the voltage. The ‘short circuit’ caused the oxidizing power due to O_2_ reacting at the cathode to be transmitted directly to the anode: as a result, and without enough H_2_ to act as counterbalance, Hyd-1 was converted rapidly to Ni–B. Power could not be restored by increasing the resistance, but momentarily connecting a second anode with active Hyd-1 resulted in immediate recovery. This effect was likened to jump-starting a car that has a flat battery, with active hydrogenases on the repairing electrode providing electrons to reactivate the hydrogenases on the fuel cell anode that have been completely converted to the Ni–B state. Volbeda and co-workers suggested that the proximity of the distal clusters in the two halves of Hyd-1 might enable a similar ‘jump-start’ reactivation of Ni–B to operate internally in the (αβ)_2_ dimer [21].

Oligomer formation can be predicted using PISA (proteins, interfaces, structures and assemblies) software which calculates energies and entropies of dissociation into monomers [24]. Table 1 shows an assessment of current [NiFe] hydrogenase structures, evaluated with Pymol and PISA. All the O_2_-tolerant hydrogenases are membrane-bound in vivo and all but the *R. eutropha* MBH show dimers of heterodimers (αβ)_2_ in the crystal structure, although it was reported that *R. eutropha* MBH may form a trimeric (αβγ)_3_ supercomplex including cytochrome *b*, based on a static light scattering analysis [25]. For O_2_-tolerant hydrogenases the free energy of dissociation (Δ*G*^diss^) within the (αβ) heterodimer is twice as large as the corresponding inter-heterodimer (αβ) values (60 vs. 30 kcal/mol), and higher than for standard hydrogenases. The hydrogenase from *A. vinosum* is the only standard hydrogenase found to crystallize as a dimer, albeit with only half the calculated Δ*G*^diss^. The distance between the two distal FeS clusters, averaging around 12.5 Å in the O_2_-tolerant enzymes, is higher in the *A. vinosum* hydrogenase, at 14 Å.

**Table 1:** Oligomeric assembly of all known [NiFe] hydrogenase structures: based on crystal structure visual assessment and PISA (proteins, interfaces, structures and assemblies) software calculations [24], the oligomeric assembly of large subunit *α* and small subunit *β* is presented along with PISA estimates of the free energy of assembly dissociation (Δ*G* ^diss^) in kcal/mol of large and small subunit within one heteromer (α:β) and between two heteromers (αβ):(αβ), where applicable

Organism (hydrogenase)	Assembly	Δ*G* ^diss^ [kcal/mol]		Distal cluster proximity/Å
		α:β	(αβ):(αβ)	
*E. coli* Hyd-1^a^	(αβ)_2_	60	32	12.2 Å Endnote library Wulff.enl
*H. marinus* MBH^a^	(αβ)_2_	64	27	12.6
*S. enterica* Hyd-5^a^	(αβ)_2_	62	32	12.7
*R. eutropha* MBH^a^	(αβ)^b^	57	–	–
*A. vinosum* Hyd	(αβ)_2_	53	16	14.0
*D. fructosovorans* Hyd	(αβ)	53	–	–
*D. gigas* Hyd	(αβ)	54	–	–
*D. desulfuricans* Hyd	(αβ)	51	–	–
*D. vulgaris* Hyd	(αβ)	57	–	–
*D. baculatum* Hyd^c^	(αβ)	53	–	–
*D. vulgaris* H Hyd^c^	(αβ)	30	–	–

The proximity of adjoining distal iron sulfur clusters was measured with the Pymol software. Organisms and enzyme PDB codes: *Escherichia coli* (3UQY), *Hydrogenovibrio marinus* (3AYY), *Salmonella enterica* (4C3O), *Ralstonia eutropha* (3RGW), *Allochromatium vinosum* (3MYR), *Desulfovibrio fructosovorans* (1FRF), *Desulfovibrio gigas* (1FRV), *Desulfovibrio desulfuricans* (1E3D), *Desulfovibrio vulgaris* (1WUL), *Desulfovibrio vulgaris* Hildenborough (2WPN), *Desulfomicrobium baculatum* (4KN9)

^a^Oxygen tolerant enzyme

^b^Described as trimeric (αβγ)_3_, including cytochromes, by Frielingsdorf et al. in gel filtration experiments but not in the crystal structure [25]

^c^[NiFeSe] hydrogenase

An interesting observation was reported recently, in which the full (αβγ)_3_ complex of *R. eutropha* MBH was studied by PFE, utilizing a tethered lipid bilayer to immobilize the enzyme on a gold electrode [26]. Unlike the normal soluble form that has been extensively studied by PFE at a graphite electrode, the (αβγ)_3_ complex did not inactivate anaerobically when poised at a high potential, and re-activation after exposure to O_2_ occurred spontaneously even at high potentials when O_2_ was removed.

The question is therefore raised—what role could extended inter-heterodimer electron transfer play in protecting a hydrogenase against O_2_? Such a protection role would represent a further example of the importance of quaternary structure in biology [27]. We have now investigated the significance of the (αβ)_2_ structure of solubilized Hyd-1 in relation to hydrogenase O_2_-tolerance. This required a systematic step-by-step strategy—establishing how to obtain the monomeric (αβ) form and separate it from the (αβ)_2_ dimer, investigating the stability and catalytic properties of the (αβ) form, comparing the O_2_-tolerances of (αβ) and (αβ)_2_, and finally devising a model that accounts for the differences.

## Materials and methods

The general procedures for obtaining purified Hyd-1 from *E. coli* cells were based on those previously established [6]. Samples were stored in liquid N_2_ as required. Size exclusion chromatography (SEC) columns were calibrated with the Sigma Aldrich MWGF1000 kit of protein standards. Since the elution volume corresponding to a certain molecular mass depended significantly on the detergent used, and varied with each repacking, the column was recalibrated each time for each detergent. Alcohol dehydrogenase (ADH) protein standard (150 kDa) was used between sample runs to help evaluate sample peak positions and confirm column integrity.

Protein samples for native electrophoresis were prepared by addition of sample buffer (Invitrogen, final concentration 50 mM BisTris, 6 N HCl, 50 mM NaCl, 10 % w/v glycerol, 0.001 % Ponceau S, pH 7.2). NativeMarkTM Unstained Protein Standard (Invitrogen) was used as a reference. The Anode Buffer consisted simply of Running Buffer (50 mM BisTris, 50 mM Tricine, pH 6.8) while the Cathode Buffer included 20× Cathode Additive (0.4 % Coomassie r G-250). The gel (pre-cast 4–16 % BisTris, Invitrogen) was run at 150 V for ca. 130 min, using the XCell SureLockTM electrophoresis system (Life Technologies). The gel was developed as follows: incubation in ca. 100 mL fixing solution (40 % methanol, 10 % acetic acid) for approx. 60 s in the microwave at ca. 700 W, followed by 15–20 min at room temperature on a gel shaker. Subsequently the gel was transferred to destaining solution (8 % acetic acid) and incubated, first in the microwave for 60 s at 700 W and then at room temperature on the shaker until satisfactory.

Protein samples for denaturing electrophoresis were prepared by addition of 4× LDS sample buffer (Invitrogen) (total volume 10 μL) and heating for 10 min at 70 °C. PageRuler^®^ pre-stained protein ladder (Thermo Scientific) was used as a reference standard. The samples were loaded onto a NuPAGEr 4–12 % Bis–Tris pre-cast gel (Invitrogen) and run at 200 V for ca. 50 min in MOPS running buffer (50 mM MOPS, 50 mM Tris base, 1 mM EDTA, 0.1 % SDS, pH 7.7) using the XCell SureLockTM electrophoresis system (Life Technologies). Gels were developed by incubation in Coomassie staining solution (0.25 % Coomassie brilliant blue, 50 % ethanol, 10 % glacial acetic acid, deionized water) for ca. 10 min at a temperature between 50 and 60 °C followed by subsequent destaining until satisfactory (20 % ethanol, 10 % glacial acetic acid, deionised water).

Procedures for isotope ratio mass spectrometry and hydrogen peroxide assays were carried out as described recently [11]. Protein film electrochemistry methods, including voltammetry and chronoamperometry were based on experiments described in the recent paper by Evans et al. [7]. All reagents used to prepare samples for solution assays or protein film electrochemistry were of analytical grade and high-purity water (Milli-Q, Millipore 18 MΩ cm) was used throughout. All gases were supplied by BOC.

## Results

### Separation of oligomeric states of Hyd-1

**Fig. 2:**
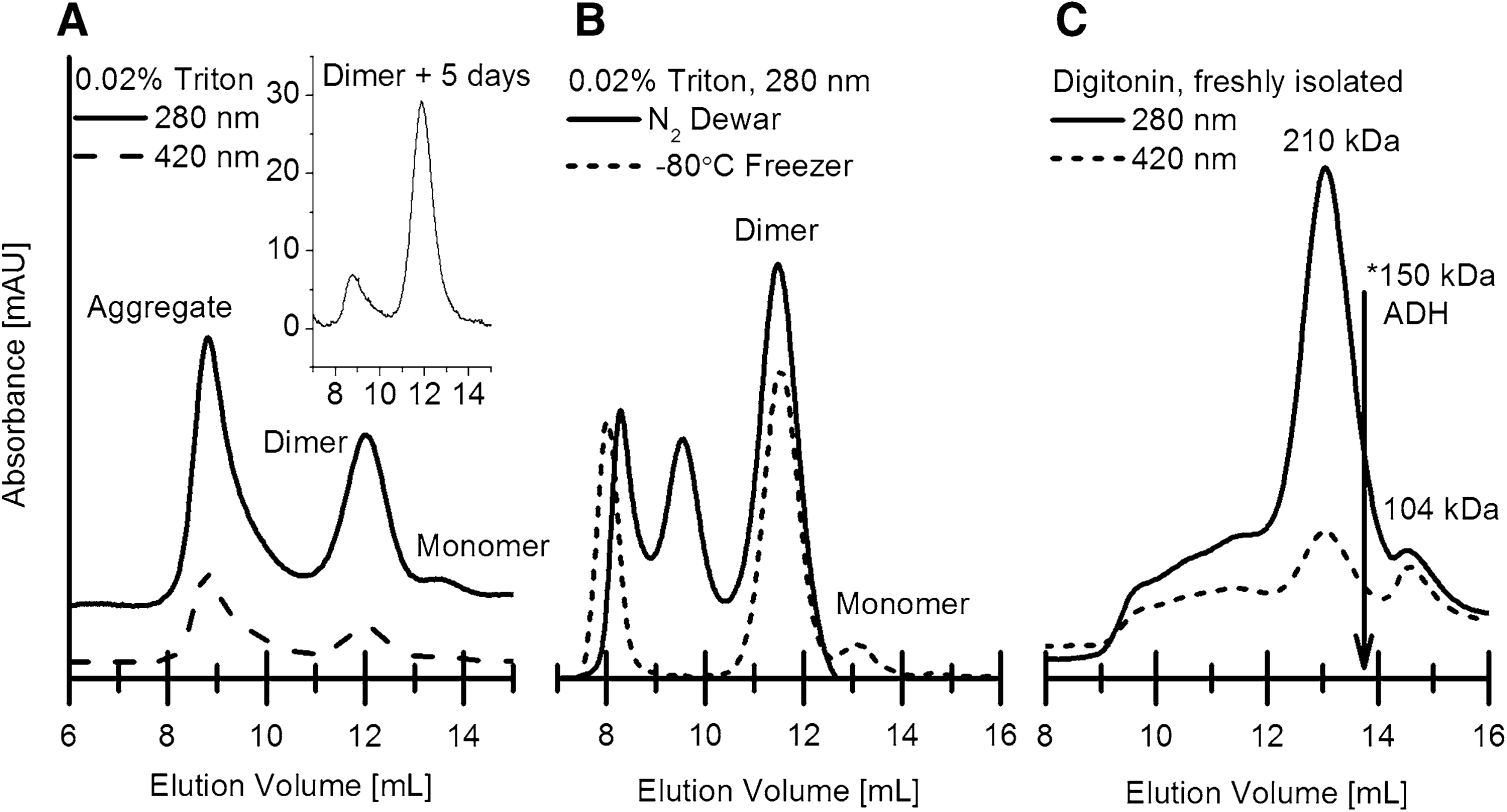
Hyd-1 size exclusion chromatography with Triton X-100 and digitonin: all samples shown were first purified by Ni–NTA affinity chromatography. The absorbance, at 420 and/or 280 nm, is shown across the elution volume. **a** Separation of Hyd-1 with Triton X-100, exchanged from Ni–NTA purification buffer (20 mM Tris, 350 mM NaCl, 0.02 % Triton; see “Materials and methods”) into running buffer prior to the experiment. The peak at 12 mL was isolated and stored at 4 °C for 5 days before being subjected to chromatography again, as shown in the *inset*. **b** Separation of defrosted Hyd-1 samples from the liquid nitrogen dewar and a −80 °C freezer, with Triton X-100. The samples were thawed immediately before applying to the column. **c** Freshly isolated Hyd-1 was incubated for 2 h with ca. 1 % digitonin before chromatographic separation in running buffer containing 0.007 % digitonin. The *arrow* and *asterisk* mark the peak position of 150 kDa alcohol dehydrogenase (ADH) protein standard. Other conditions: flow rate 0.15 mL/min, superdex 200 HR column (10/30), pH 7.0, 50 mM sodium phosphate, 0.10 M NaCl, 4 °C. The column was repacked between experiments **a**, **b** and **c**

**Fig. 3:**
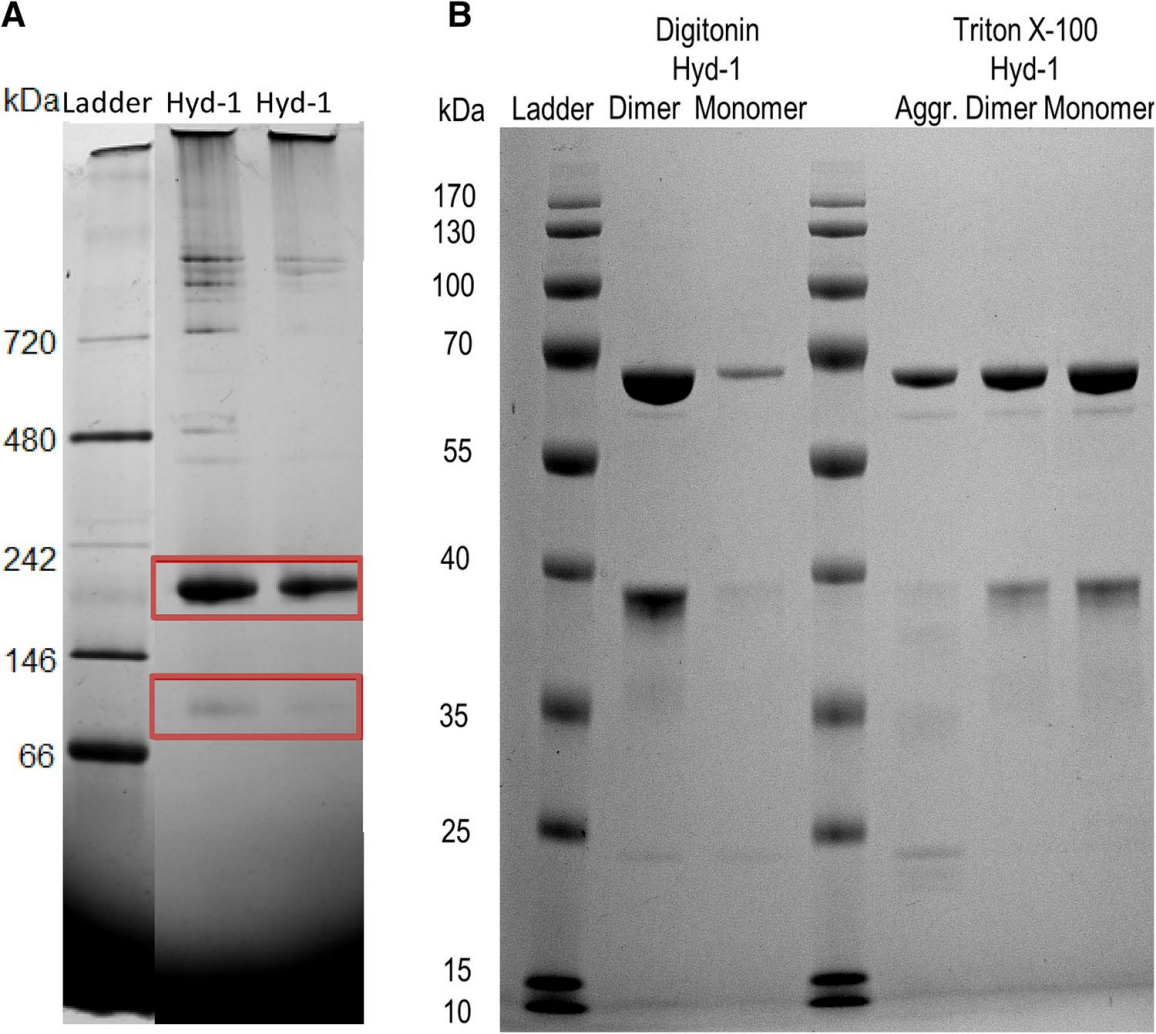
Native and SDS polyacrylamide gel electrophoresis of Hyd-1 samples: **a** native PAGE of isolated full Hyd-1 WT, **b** SDS PAGE of separated Hyd-1 (WT) fractions. Non-relevant lanes have been omitted

Figure 2a shows a Hyd-1 oligomer separation carried out in buffer containing 0.02 % Triton X-100. Soluble aggregate is found near the void (around 8–9.5 mL). A large, well-defined peak at 12 mL and a much smaller peak at 13.5 mL, corresponding to molecular masses of approximately 220 and 110 kDa, respectively, are assigned to dimer and monomer forms of Hyd-1. Significant absorbance at 420 nm due to iron-sulfur clusters and heme is found for both aggregate and the dimer peaks. The inset (Fig. 2a) shows an SEC analysis of the dimer fraction after 5 days storage at 4 °C. The fact that most protein elutes at the same volume (12 mL) suggests that the dimeric form is the stable state. However, the reproducibility of the separation experiment with Triton X-100 (major panel Fig. 2a) was poor, especially with regard to the monomer, the low yield of which is clear from analysis of the isolation products by Native gel electrophoresis, as shown in Fig. 3a. The elution profile depended further on how isolated Hyd-1 is stored. Replacing Triton X-100 with digitonin, a detergent used to purify *R. eutropha* membrane-bound hydrogenase (MBH) in a fragile multimeric complex with its native cytochrome *b*_562_, led to more reliable isolation of Hyd-1 monomer [25]. The best results were obtained by pre-incubating freshly purified samples in buffer containing ca. 1 % (wt/vol) digitonin then using just 0.007 % digitonin in the running buffer to minimize detergent precipitation. The molecular masses of the protein peaks identified as ‘Dimer’ and ‘Monomer’, calculated relative to a full calibration, are 210 kDa and 104 kDa, respectively. Particularly notable is the presence of two distinct A_420_ peaks pairing with the A_280_ dimer and monomer peaks.

To evaluate the subunit composition of the aggregate and putative dimer and monomer peaks isolated from Triton X-100 and digitonin SEC experiments (Fig. 2b/c), denaturing electrophoresis was carried out (Fig. 3b). All samples show characteristic large (64.6 kDa) and small (36.8 kDa) subunit bands just below the 70 and 40 kDa marker bands, demonstrating that the monomer and dimer fractions (at approximately 110 and 220 kDa total mass) are structurally intact and consist of at least one and two assemblies, respectively, of a large and small subunit each. Some lanes are slightly overloaded (widening of the large subunit band) which is somewhat unavoidable in the search for fainter bands. While the overloading prevents a truly reliable quantitative comparison of band density, the Triton X-100 aggregate appears to have a particularly low proportion of small subunit.

### Reactivation and O_2_ reduction in solution

Hydrogen oxidation by hydrogenases in solution is easily measured by monitoring the increase in absorbance at 604 nm (*A*_604_) due to enzymatic reduction of benzyl viologen in the presence of H_2_ [6]. When using enzyme samples that have previously been activated under H_2_ then exposed to air to cause inactivation, a lag phase in which there is no change in *A*_604_ is commonly observed at the beginning of the assay, when all the viologen is in the oxidized state. The lag phase arises because enzyme reactivation depends on the supply of electrons (transferred to viologen) originating from H_2_ oxidation by those sites that are active: there are no electrons available at the start of the experiment, and few active enzyme molecules to generate them.

The lag phases of dimer and monomer samples were compared. Initial hydrogen oxidation assays indicated that dimer samples were more than twice as active as monomer, based on protein concentrations determined by Bradford assay. However, the Bradford assay is very susceptible to, and easily biased by, the presence of detergents, that are essential for sample preparation, storage and use. Reagent binding might also be significantly affected by the level of protein aggregation. Determination of protein concentration by UV–vis spectroscopy (*A*_280_) yielded turnover rates that were closer in value. To compare different measurements in the light of this uncertainty, the amount of enzyme used in the eventual solution-based reactivation assays was adjusted to yield samples of equal final activity and not apparent protein concentration.

**Fig. 4:**
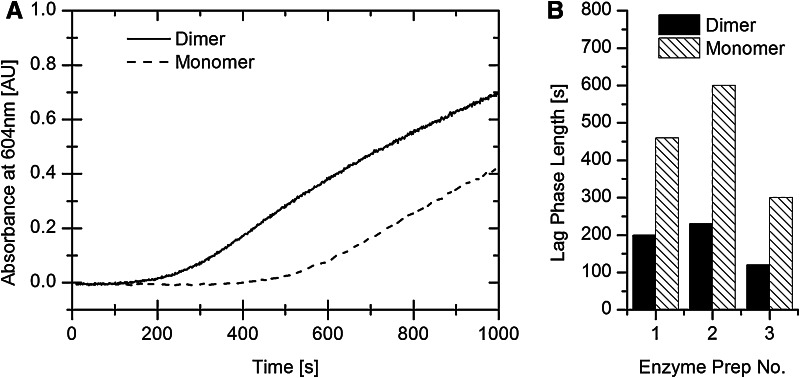
Benzyl Viologen reduction lag phase assay: **a** reduction of benzyl viologen (*ε*_604_ = 9.82 mM^−1^ cm^−1^ for reduced form) monitored over time for Hyd-1 dimer and monomer solutions. Conditions: H_2_-saturated buffer, pH 7.0, 50 mM sodium phosphate, 0.10 M NaCl, 0.01 % digitonin, room temperature. **b** Comparisons of the length of the lag phase for three separate experiments with enzymes from three separate preparations. The time point of the end of the lag phase was determined by eye

Figure 4a shows a typical result of the lag phase experiment, in which previously activated dimer and monomer samples were exposed to air for several hours before the assay. Immediately after injection into H_2_-saturated benzyl viologen buffer, the absorbance at 604 nm was recorded over time. The eventual rate of viologen reduction in the samples was adjusted to be as similar as possible; in this case approx. 5.9 μM s^−1^ for the dimer and 6.7 μM s^−1^ for the monomer solution. The lag phases differ greatly between monomer and dimer. Figure 4b compares the lag phases observed in three sets of experiments, carried out using enzyme from three separate preparations. Although both overall activity and length of lag phase vary between different Hyd-1 preparations, the difference between dimer and monomer for each individual preparation was clear and consistent: the lag phase of the monomer is always slightly more than twice as long as that of the dimer.

**Fig. 5:**
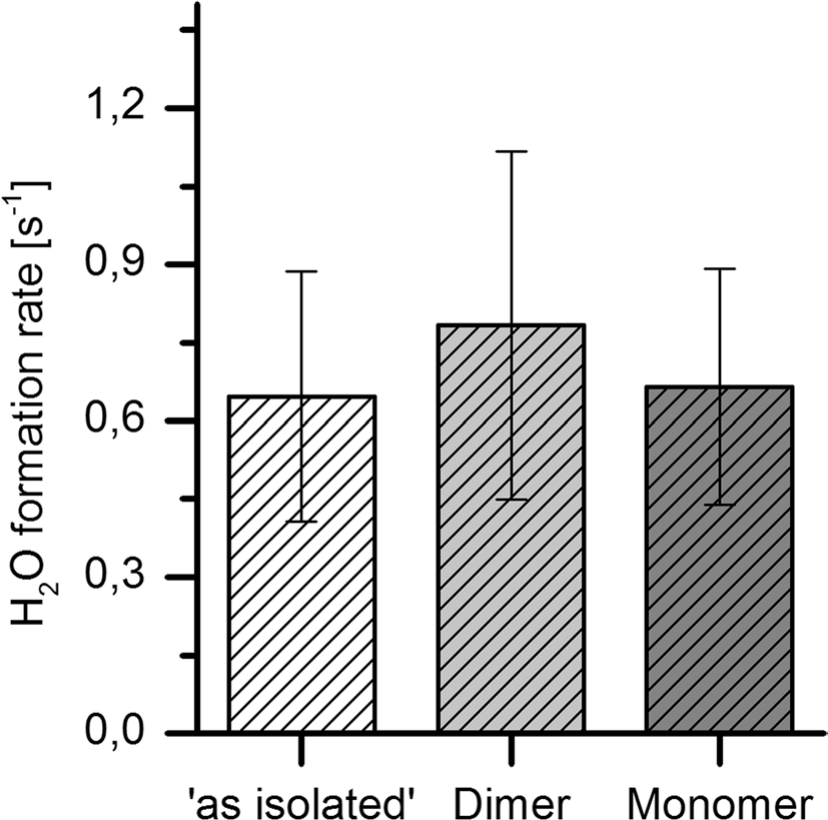
Rate of formation of H_2_^18^O by dimer and monomer forms of Hyd-1: the rate of water formation (μM H_2_^18^O per μM Hyd-1) compared to blank controls is given for `as isolated’ Hyd-1 and resolved dimer and monomer forms. The *error bars* show the standard deviation across 18, 3 and 3 measurements, respectively. The concentration of H_2_^18^O was measured by isotope ratio mass spectrometry as described previously [11]. The experiments were conducted under an atmosphere of 90 % H_2_ and 10 % ^18^O_2_ at pH 7.0 and 20 °C

Oxygen-18 reduction experiments for dimer and monomer samples were carried out using isotope ratio mass spectrometry to detect the amount of H_2_^18^O formed. The methodology was as described recently [11]. Due to the limited amount of monomer available, the following protocol was used. First, the concentration of dimer samples was determined by Bradford assay, then the absorbance at 280 nm (A_280_) was recorded by UV–vis spectroscopy to obtain a molar absorption coefficient from which the concentration of monomer samples could also be determined. The concentration of enzyme is expressed with reference to the functional monomer containing one active site and having a mass of 101 kDa. Figure 5 shows the results obtained from eighteen data points in Ref. [11] where an H_2_^18^O formation rate of 0.65 s^−1^ was obtained; this compares with rates obtained (three datum points) from samples incubated for 4, 10 and 14 h for dimer (0.78 s^−1^) and monomer (0.67 s^−1^). Long incubation times were needed to accumulate enough products for mass spectrometry measurements, due to Hyd-1 monomer concentrations being as low as 0.06 μM.

### Protein film electrochemistry of dimer and monomer

To evaluate any obvious electrochemical differences between dimer and monomer forms of Hyd-1, cyclic voltammetry was performed on enzyme films grown using the two different fractions on a pyrolytic graphite edge (PGE) electrode using published procedures [3].

**Fig. 6:**
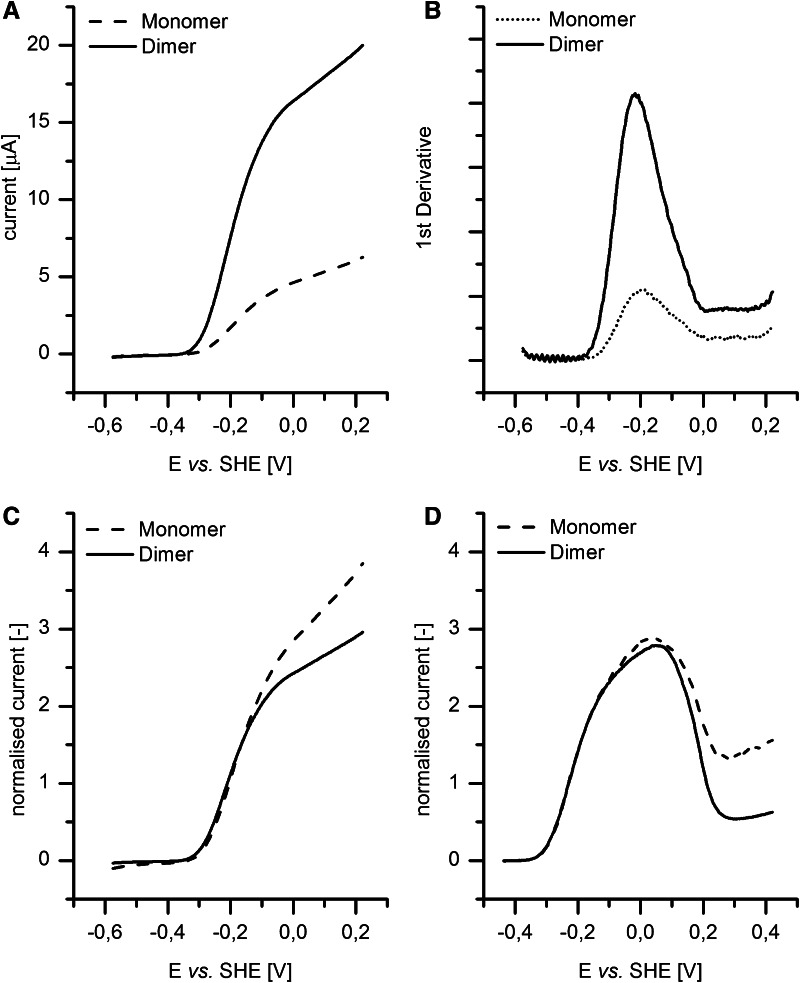
Protein film voltammetry of dimer and monomer forms of Hyd-1: **a** averaged forward and backward scans (scan rate 1 mV s^−1^) of dimer and monomer films on a rotating PGE electrode. **b** First derivative of the averaged cyclic voltammograms from *panel* **a**. **c** Voltammograms normalized with respect to the current at the potential of the maximum derivative. **d** Linear sweep scans of dimer and monomer films, slowly swept from +0.42 to −0.44 V vs. SHE at 0.1 mV s^−1^, to monitor reactivation of the anaerobically inactivated oxidized state (Ni–B). As in *panel* **c**, the voltammograms are normalized to the current at the potential of maximum derivative. Common conditions: 100 % H_2_, pH 6.5, electrode rotation rate 3000 rpm

Figure 6a shows cyclic voltammetry experiments carried out with monomer and dimer forms. The potential was scanned at 1 mV s^−1^ and the PGE electrode was rotated at 3000 rpm, conditions found to give steady-state voltammetry free of time dependence or mass-transport limitations. The line shows the average current for forward and reverse scans. The dimer yielded significantly higher catalytic current than the monomer (an approximate fourfold difference being typical) irrespective of whether the film was obtained through application of more or equally concentrated dimer solution relative to the monomer. Only in experiments with very dilute monomer samples (a symptom of very low monomer yields in early isolation experiments) were larger differences in total current observed. The plot in Fig. 6a initially suggests that the monomer has a more positive onset potential (the potential at which the catalytic current begins to rise) so a normalization procedure was devised to allow a better comparison of the two results. Figure 6b compares the first derivative (slope) of the monomer and dimer voltammograms, which locates a characteristic potential at which the rate increases most steeply. The potentials of maximum rate of current change are very similar for monomer and dimer, at approximately −203 and −212 mV vs. SHE, respectively. By normalizing the voltammograms to the currents at these potentials, Fig. 6c was obtained: importantly, H_2_ oxidation by both oligomeric forms of Hyd-1 shows a nearly identical response at the low-potential edge where both show the same onset potential of approx. −335 mV vs. SHE at the conditions used. With the H_2_/H^+^ equilibrium potential *E*_eq_ at −391 mV vs. SHE, the onset overpotential requirement is approximately 56 mV for both oligomeric forms.

At higher potential however, the two voltammograms differ significantly. Three factors principally affect the catalytic current of Hyd-1 at high potentials: (1) At sufficiently high currents, the current could be limited by H_2_ mass transport; this factor was eliminated by the use of 1 bar H_2_ and a rotation rate of 3000 rpm (after establishing that a higher rotation rate did not result in an increase in current). (2) Dispersion of interfacial electron-transfer rates, due to the various orientations that can be adopted by enzyme molecules on an electrode surface, gives rise to a residual current instead of the flat plateau that is normally expected once electron transfer is no longer limiting [28]. (3) Slow oxidative conversion of the enzyme into the inactive Ni–B state. A procedure was devised to assess the potential at which Ni–B is stable. The Hyd-1 film on the electrode was subjected to a high-potential poise (+0.42 V vs SHE) for sufficient time to convert much of the sample to Ni–B, then the potential was scanned in the negative direction at a very low scan rate, e.g. 0.1 mV s^−1^. In such an experiment, shown in Fig. 6d, the data were normalized in the same way as those in panel C, and the two traces overlay very well at low potential. Although the two voltammograms differ at high potential because the fraction of monomeric enzyme that has inactivated is smaller (the extent of anaerobic, electrochemical inactivation may depend on the proficiency of electronic coupling between enzyme and electrode) the re-activation potential referred to as *E*_switch_ (an empirical reference point for the re-activation process) is altered very little.

The hallmark of oxygen tolerance is sustained activity in the presence of O_2_. Mass spectrometry results have shown that Hyd-1 achieves this through rapid reduction of O_2_ to water, which results only in Ni–B [11]. Chronoamperometric experiments on Hyd-1, in which the H_2_ oxidation current at a given potential was monitored during each addition and subsequent removal of O_2_ from the gas supply, showed that for each O_2_ concentration, the current settled at a new stable level, equivalent to O_2_ behaving as a reversible inhibitor [7]. A simple term for the fractional (steady-state) activity *f* was introduced, *f* being the steady-state H_2_ oxidation current for a given O_2_ level relative to that observed in the absence of O_2_. It was proposed that *f* depends on the rates of inactivation *r*_I_ and reactivation *r*_A_ to/from the Ni–B state according to Eq. 1:

1 $$f = \frac{\text{Active enzyme}}{{{\text{Total enzyme}} }} = \frac{{r_{\text{A}} }}{{r_{\text{A}} + r_{\text{I}} }}.$$

**Fig. 7:**
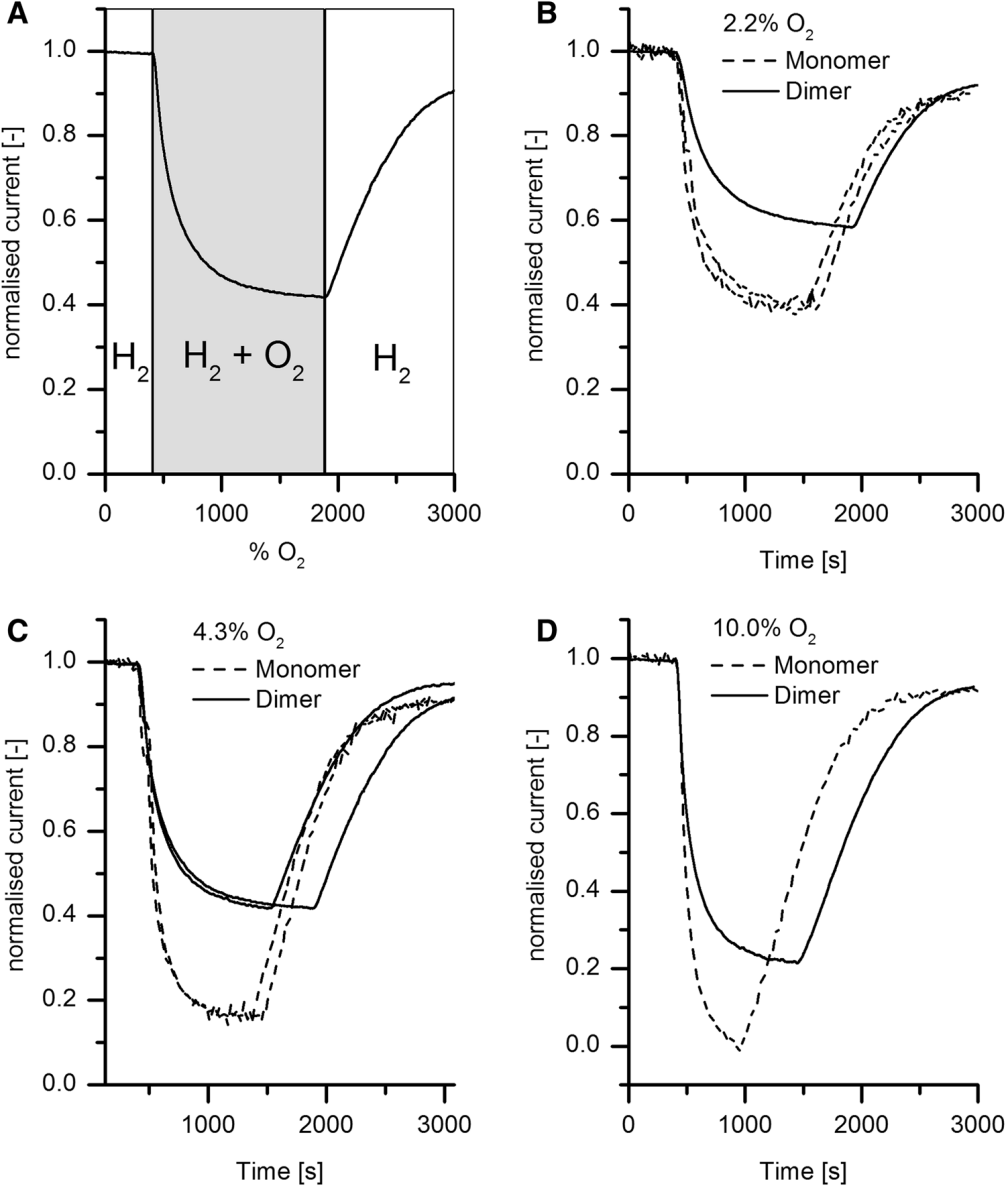
Chronoamperometric experiments of H_2_ oxidation by dimer and monomer forms of Hyd-1 under increasing concentrations of O_2_. **a** General scheme showing how the H_2_ oxidation current of Hyd-1 dimer and monomer films was monitored at 10 mV vs. SHE as the gas mixture is changed. The current is normalized with respect to the stable current under 100 % H_2_ After 400 s O_2_ is added to the gas stream and removed again once the current has settled at a new stable level. The compositions of the O_2_/H_2_ atmospheres were: **b** 2.2 % O_2_, 97.8 % H_2_; **c** 4.3 % O_2_, 95.7 % H_2_; **d** 10.0 % O_2_, 90.0 % H_2_. Prior to each experiment the protein films on rotating PGE electrodes were activated at −0.55 V vs. SHE and allowed to stabilize. Common conditions: pH 7.0 electrode rotation rate 2500 rpm

Chronoamperometry experiments were carried out to determine how the oligomeric state of Hyd-1 affects the steady-state activity observed in the presence of different amounts of O_2_. The experiment was conducted at 10 mV vs. SHE, a potential sufficiently high to have a good H_2_ oxidation current but, equally, negative enough to avoid anaerobic Ni–B formation. In Fig. 7a, the H_2_ oxidation rate under 100 % H_2_ was allowed to stabilize for 400 s, providing the reference current by which the individual experiments could be normalized. Then O_2_ was introduced into the gas stream and the decreasing current was monitored for a period of time before the atmosphere was restored to 100 % H_2_. Panels B–D in Fig. 7 show the results of the H_2_/O_2_ experiment for monomer and dimer at different O_2_ concentrations. Although the H_2_ concentration varies from 100 to 90 %, this small range does not affect the current as these levels are far above the Michaelis constants which lie in the region of 1 % H_2_. The resulting catalytic current at each O_2_ addition is always lower for monomer. The currents for both dimer and monomer settle at new steady-state values for 2.2 and 4.3 % O_2_, but the monomer is unable to sustain H_2_ oxidation at 10 mV vs. SHE in the presence of 10 % O_2_ and activity decreases to zero. In all cases however, restoring 100 % H_2_ results in rapid recovery, showing that inactivation is easily reversed, as expected if only Ni–B is formed: hence monomer, like dimer is not converted into Unready states under these conditions.

## Discussion

Use of freshly isolated Hyd-1 and digitonin proved not only to allow the clearest, most reliable separation of distinct dimer and monomer fractions, but also produced the least wastage in the form of aggregate. Although initially stable and soluble, aggregate fractions were observed to give substantial precipitate over time. Obtaining sufficient quantities of monomer was the chief challenge for a rigorous characterization of the monomer fraction, since only the trailing side of each monomer peak could be used to avoid isolating a heterogeneous sample. Applying very concentrated samples to a gel filtration column, in an effort to reduce the number of purifications needed to accumulate enough enzymes to study, tended to be futile, since loading concentrated samples lowers the peak separation.

The (αβ:αβ) dissociation free energy of 31.8 kcal/mol (Table 1) for Hyd-1 is likely an overestimate since the underlying calculation does not account for the presence of detergent. From gel electrophoresis, both dimer and monomer as well as larger aggregate fractions consist of both small (36.8 kDa) and large (64.6 kDa) subunits. The ca. 210 and 104 kDa oligomers are thus confidently assigned as dimer (αβ)_2_ and monomer (αβ).

The onset overpotential of approximately +56 mV (pH 6.5, 100 % H_2_ and 30 °C) and the current response to increasing potential around the onset potential are similar for both monomer and dimer. Onset overpotential requirements for `as-isolated’ Hyd-1 were previously reported as approximately +50 mV under 10 % H_2_ (pH 6.0, 30 °C) by Lukey et al. [6], or in a more detailed study by Murphy and co-workers as +54 mV and +82 mV at pH 6.0 and pH 7.0, respectively (100 % H_2_, 37 °C) [4]. The values determined here for the pure oligomeric fractions agree well with these earlier measurements, noting that the onset potential increases with decreasing pH [4].

Differences in the voltammetry traces recorded for dimer and monomer are most prominent at high potential. The more pronounced residual current (slope) for monomer may reflect the ability of the (newly) solvent-exposed area in the monomer (i.e. the interface area in the dimer) to offer a greater range of interactions and orientations with the electrode surface. Importantly, the potential *E*_switch_, a measure of the stability of Ni–B, is unchanged, and in conjunction with the similar onset overpotential, supports the idea that the Hyd-1 monomer is fully functional.

A particularly striking difference between monomer and dimer forms of Hyd-1 was displayed in the non-electrochemical, solution assays of H_2_ oxidation, where it was established that the initial lag phase for the monomer is more than twice as long as for the dimer (Fig. 4b). Questions arising are: (1) does the dimeric organization help in ensuring exclusive formation of rapidly reactivated Ni–B, and does the monomer therefore produce less-easily re-activated Unready states (Ni–A) ?. We noted earlier that Hyd-1 activity in solution is maintained at a constant level in 10 % O_2_, an observation that argues against Ni–A formation [11]. (2) Does the dimeric organization affect the rates of reactivation of inactive states? (3) Can a result similar to the difference in lag phase be reproduced in a more controlled environment, where uncertainty over enzyme concentration is not important? The chronoamperometry experiments, in which Hyd-1 attains a steady state with simultaneous substrate (H_2_) and competing substrate/inhibitor (O_2_) turnover, were helpful in answering these questions.

The facts that both dimer and monomer attain a steady-state H_2_ oxidation activity in the presence of O_2_ (Fig. 7) and recover activity fully when O_2_ is removed show that the ability to exclusively form Ni–B (and no Ni–A) and thus reduce O_2_ completely to water are unaffected by the oligomeric state of the enzyme. Formation of even a small fraction of Ni–A would lead to a persistent and largely irreversible decrease in catalytic current. Even under 10 % O_2_ the almost zero activity of the monomer recovers virtually completely when the O_2_ is removed from the gas stream. The main difference in all cases is that the fractional activity is significantly diminished for the monomer compared to the dimer. Further analysis of these steady-state levels holds the key to developing a plausible mechanistic explanation of the effects of dimerization.

Using the steady-state description introduced above, rates are substituted by rate constants: and assuming *r*_I_ = *k*_I_[O_2_] at the low [O_2_] values used (Evans et al. measured the initial rate of O_2_ reaction with active Hyd-1 and found it to increase linearly with O_2_ concentration [7]), *f* at any given potential is given by:

2 $$f = \frac{\text{Active enzyme}}{\text{Total enzyme}} = \frac{{k_{\text{A}} }}{{k_{\text{A}} + k_{\text{I}} [{\text{O}}_{2} ]}}$$

**Fig. 8:**
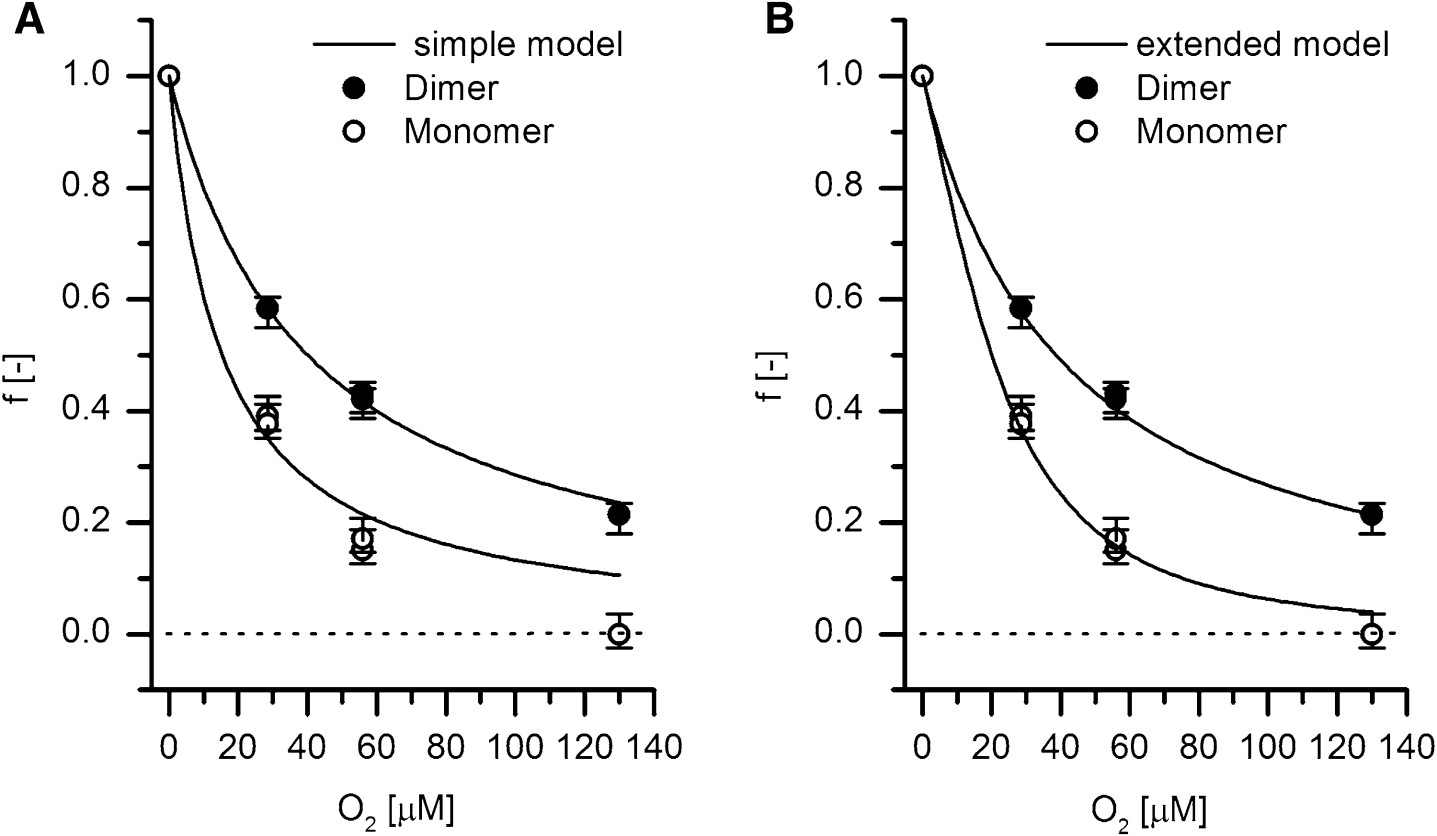
Steady-state activities of dimer and monomer forms of Hyd-1, relative to initial activity, as a function of O_2_ concentration. The respective values were obtained from the chronoamperometry experiments shown in Fig. 7b–d. *Error bars* represent estimated uncertainty based on film loss, residual slope and electrical noise contributions. The zero current line is drawn as a *dotted black line* for reference. **a** *Solid lines* represent the fit to the simple model of O_2_ catalysis by Hyd-1 in the presence of H_2_ according to Eq. 2, as explained in the main text. **b** *Solid lines* represent the fit to the extended model (Eq. 4) based on oligomeric assembly, oxidative side reactions and electron transfer

Figure 8a shows a fit to Eq. 2 for the fraction of active dimer at each O_2_ concentration. The fit used a ratio *C* of apparent reactivation to inactivation rate constant of *C*_D_*/*μM = *k*_A_*/k*_I_ = 40 (at +10 mV). For the monomer, no fit could be obtained with any combination of rate constants; the best approximation was *C*_M_*/*μM = *k*_A_*/k*_I_ = 15 with *k*_A_ being 38 % that of the dimer.

Although the individual rate constants cannot be obtained directly from these experiments, previous experiments with Hyd-1 provide a guideline. Wulff et al. determined a rate constant of *k*_I_ = 0.002 (μM O_2_)^−1^ s^−1^ at 20 °C [11] while Evans et al. found *k*_I_ = 0.0038 (μM O_2_)^−1^ s^−1^ at 30 °C [7]. From an Arrhenius plot, the rate constant for inactivation at 25 °C is estimated as *k*_I_ = 0.0028 (μM O_2_)^−1^ s^−1^. With *C*_D_*/*μM = *k*_A_*/k*_I_ = 40 (see above) the rate constant for reactivation is deduced to be *k*_A_ = 0.112 s^−1^ for the presented fit to the dimer data. An interesting point, however, is that *k*_A_ is strongly potential dependent (see below). Since the reactivation rate is very fast at low potentials, it is not directly measurable under experimental conditions chosen to allow significant H_2_ oxidation activity at high O_2_ concentrations. This problem was addressed previously by Evans et al. [7] by extrapolation from data obtained at both higher potential and lower temperature to the desired conditions, with the help of electrochemical activation and Arrhenius plots. Using the same process, rate constants of *k*_A_= 0.136 s^−1^ and *k*_A_= 0.186 s^−1^ were calculated for −10 mV vs. SHE and 25 °C from two separate data sets in the paper by Evans et al.; the discrepancy between these two values illustrates the uncertainty and deviations introduced by double extrapolation. These calculations showed that the fit to the dimer data is achieved with rate constants that agree reasonably well with predicted values.

**Scheme 1:**
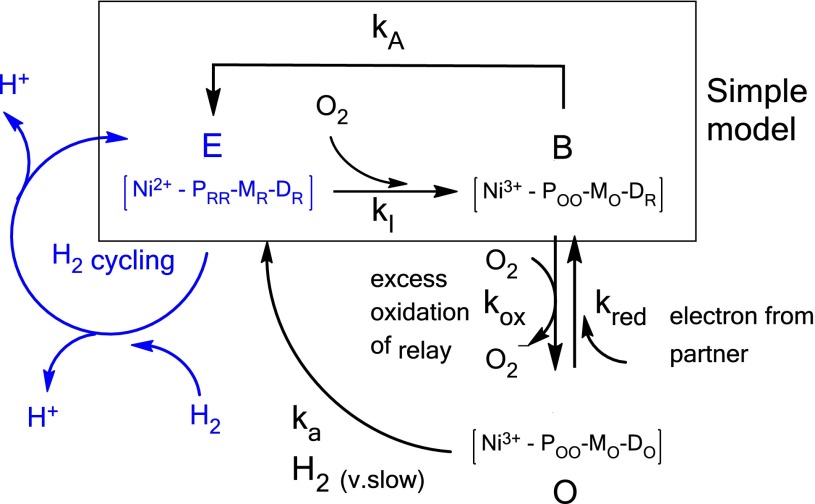
Models for the reactions of Hyd-1 with O_2_: the simple model, enclosed in the *rectangle*, assumes rapid reactivation of the enzyme by intramolecular electron transfer. The *blue text* represents enzyme that is active in H_2_ oxidation. Note that formation of E from B may also require the presence of H_2_ [29]

The simple model shown in Scheme 1 assumes that O_2_ attacks the active enzyme (E) when the active site is in the highest active oxidation state and all the FeS clusters are reduced. In the Ni–B state (B) that is rapidly formed, three electrons have been transferred from the FeS relay leaving one site (the distal cluster) still reduced. State B can be reactivated rapidly, starting with reduction of the active site Ni back to Ni^2+^ by intramolecular electron transfer, but the fact that *k*_A_ is a potential-dependent rate constant suggests that we should regard the FeS clusters of the relay collectively as acting like a low-level tunneling barrier—a ‘wire’ along which electron transfer is accelerated by a potential difference. The enzyme now re-enters the catalytic H_2_ cycle through binding of H_2_ and subsequent turnover [29]. Binding of H_2_ to the active site in the Ni^2+^ state to re-establish E is assumed to occur on a much faster time scale than the relatively slow reaction with O_2_, and is thus considered to be effectively instantaneous. After an indefinite number of H_2_ turnovers, another O_2_ may attack, and active enzyme E may enter another cycle of the slower O_2_ catalysis.

The simple model for the reaction of Hyd-1 with O_2_ was now reconsidered to see if any improvement could be made. An extension of the model needed to account for the observation that the active monomer fraction decreases more strongly than the active dimer fraction when the O_2_ concentration is increased; hence two further mechanistic features were introduced. First, it had been noted previously that a relatively small but significant superoxide/peroxide producing side reaction is observed for Hyd-1 in the presence of O_2_ [11]. Second, we considered the likelihood that the two partners in a dimer might be able to share electrons via the distal clusters. These considerations yielded the extended reaction scheme included in Scheme 1.

The first stage ocurs as before: O_2_ attacks an active enzyme molecule E to give Ni–B (B in Scheme 1) in a four-electron reaction that produces two molecules of water. If we assume that all the FeS clusters are reduced before O_2_ attacks, two electrons are delivered from the proximal FeS cluster P, one stems from the medial cluster M and the final electron results from oxidation of the active site Ni, such that the distal cluster D can remain reduced. Protons are omitted from this scheme which also aids simplicity. Under steady-state conditions B is best described as (Ni^3+^-P_OO_M_O_D_R_). Formation of a Michaelis complex E:O_2_ in the initial reaction of active enzyme with O_2_ was also considered but discarded, as a fit to the data simply required a very large *K*_m_ value to impose the same linearity as in the simpler model.

The extended model includes the possibility that inactive enzyme B may react in an alternative manner and become more oxidized, exhausting the FeS relay system of electrons. The electron residing in the FeS relay system that is normally available to transfer to the active site could transfer instead to another O_2_ molecule. Such a site is likely to be the distal cluster that lies closest to the protein surface and able to undergo an outer-sphere reduction of O_2_ to superoxide O_2_^−^, resulting in a fully oxidized enzyme O that requires external electrons for re-activation. The source of this electron depends on the particular experiment (electrochemical vs solution), whether monomer or dimer is present, and on the rate constant *k*_red_ for electron transfer to the distal cluster, specifically, either between subunits (*k*_DD_) or interfacial (*k*_ED_). It is very likely that the electron must tunnel over a longer distance from the electrode to the distal cluster than between the distal clusters, so that *k*_DD_ ≫ *k*_ED_. Thus, even on an electrode the dimer should have an advantage in O_2_ tolerance, since at any point in time it is likely to have at least one reduced distal cluster. Superoxide formation at the distal cluster could result in damage, although in the electrochemical experiment, O_2_^−^ would be removed rapidly by electrode rotation.

Another possible pathway for reactivation is the very slow reaction (*k*_a_) of fully oxidized enzyme O with H_2_ to activate the enzyme, a process that accounts for the lag period in solution assays when no external electron donor is present. There is one precedent for this possibility, in which slow, direct reaction of Ready (Ni–B) with H_2_ is reported [30]. In the electrochemical experiment, when compared to reduction of the distal cluster by long-range electron transfers, *k*_a_ should be largely irrelevant for the dimer; hence, the overall rate of (dimer) reactivation *v*_A_ from B to E can be simplified by analogy to a reversible inhibition (Eq. 3) depending on [O_2_] with an apparent inhibition constant *K*_C_ proportional to *k*_red_*/k*_ox_:

3 $$v_{\text{A}} = \frac{{k_{\text{A}} }}{{1 + \frac{{[{\text{O}}_{2} ]}}{{K_{\text{C}} }}}}.$$

Incorporation of *v*_A_ in place of *k*_A_ in Eq. 2 yields the fraction of active enzyme for the new extended model:

4 $$f = \frac{\text{Active enzyme}}{\text{Total enzyme}} = \frac{{k_{\text{A}} \left( {1 + \frac{{[{\text{O}}_{2} ]}}{{K_{\text{C}} }}} \right)^{ - 1} }}{{k_{\text{A}} \left( {1 + \frac{{[{\text{O}}_{2} ]}}{{K_{\text{C}} }}} \right)^{ - 1} + k_{\text{I}} [{\text{O}}_{2} ]}}.$$

Incorporating this extension into the model improves the fit to the data at high O_2_ concentration even for the dimer (see Fig. 8b). The large value for *K*_C_ used for the dimer (*K*_C_ = 1000) implies that distal cluster reduction via the distal–distal pathway is very fast compared to *k*_ox_, so that the oxidizing side reaction has a small effect which only becomes discernible at high O_2_ concentrations (where coinciding oxidation of both distal clusters in a dimer also becomes more probable). In the monomer, oxidation of the distal cluster in state B leaves only the slower *k*_ED_ contribution to *k*_red_ and a much lower value for the constant *K*_C_ is expected. The slowest pathway for reactivation *k*_a_ might also play a small role under these circumstances. In agreement with these considerations, a good fit to the monomer data is obtained using the new extended model with *K*_C_ = 20 as shown in Fig. 8b.

Two further points are worth noting. The potential-dependence of the observed *k*_A_ values [7] is also consistent with the effect of electrode potential on distal cluster oxidation state and reverse supply of electrons *k*_ED_ for reactivation, as discussed above. The isotope ratio mass spectrometry experiment also supports the idea that the differences in O_2_ tolerance between dimer and monomer do not originate from separate pathways for primary O_2_ attack (*k*_I_).

The excellent fit of the new extended model to the data for both dimer and monomer (Fig. 8b) is a strong argument for its validity. On a statistical basis, attacks by O_2_ molecules during normal H_2_ turnover are likely only to affect one half of the enzyme at a given instant unless the O_2_ concentration is very high. Rapid sharing of electrons between distal clusters for delivery to the active site, as described by the extended model, also explains why the lag phase in solution assays is much longer for monomer. According to Scheme 1, the lag phase is due to the very slow direct reaction with H_2_ (*k*_a_). For the dimer, only half of all active sites need to be reactivated via this slow pathway, explaining the observed ca. two fold difference in lag phase between dimer and monomer (Fig. 4). Once enzymes have become activated, electrons are available through the build up of reduced viologen and the process accelerates.

In conclusion, we have demonstrated that it is possible to separate distinct oligomeric states of *E. coli* Hyd-1. Specifically, an (αβ)_2_ dimer of heterodimers and an (αβ) monomer of heterodimers were isolated. The dimer is very stable, easy to isolate and is the favored species at increasing detergent concentrations where larger aggregate fractions are broken down. Experiments carried out to investigate the functional advantages that a dimer structure confers led to increased O_2_ tolerance as being the most significant property. The mechanism is complicated but we have proposed that O_2_ tolerance depends in some way on the ability to transfer electrons between the distal clusters in each (αβ) monomer half. Just as the explanation for the fuel cell experiment lay in an analogy with jump-starting a car with a flat battery, the normal function of Hyd-1, which is to catalyze H_2_ oxidation in the face of regular attacks by O_2_, depends upon the constant presence of a partner to provide a rescue electron when needed. Electron transfer between each half of the dimer is more rapid than interfacial electron transfer at modest electrochemical driving force. In conclusion, teamwork pays off even in biological electron transfer!
